# Intracellular Accumulation of Linezolid and Florfenicol in OptrA-Producing *Enterococcus faecalis* and *Staphylococcus aureus*

**DOI:** 10.3390/molecules23123195

**Published:** 2018-12-04

**Authors:** Yingyu Wang, Xiaowei Li, Yang Wang, Stefan Schwarz, Jianzhong Shen, Xi Xia

**Affiliations:** 1 Beijing Advanced Innovation Center for Food Nutrition and Human Health, College of Veterinary Medicine, China Agricultural University, Beijing 100193, China; wangyingyu_1992@163.com (Y.W.); xiaowei@cau.edu.cn (X.L.); wangyang@cau.edu.cn (Y.W.); sjz@cau.edu.cn (J.S.); 2 Institute of Microbiology and Epizootics, Centre for Infection Medicine, Department of Veterinary Medicine, Freie Universität Berlin, 14163 Berlin, Germany; stefan.schwarz@fu-berlin.de

**Keywords:** linezolid, florfenicol, *Enterococcus*, *Staphylococcus*, active efflux, ABC transporter

## Abstract

The *optrA* gene, which confers transferable resistance to oxazolidinones and phenicols, is defined as an ATP-binding cassette (ABC) transporter but lacks transmembrane domains. The resistance mechanism of *optrA* and whether it involves antibiotic efflux or ribosomal protection remain unclear. In this study, we determined the MIC values of all bacterial strains by broth microdilution, and used ultra-high performance liquid chromatography-tandem quadrupole mass spectrometry to quantitatively determine the intracellular concentrations of linezolid and florfenicol in *Enterococcus faecalis* and *Staphylococcus aureus*. Linezolid and florfenicol both accumulated in susceptible strains and *optrA*-carrying strains of *E. faecalis* and *S. aureus.* No significant differences were observed in the patterns of drug accumulation among *E. faecalis* JH2-2, *E. faecalis* JH2-2/pAM401, and *E. faecalis* JH2-2/pAM401+*optrA*, but also among *S. aureus* RN4220, *S. aureus* RN4220/pAM401, and *S. aureus* RN4220/pAM401+*optrA*. ANOVA scores also suggested similar accumulation conditions of the two target compounds in susceptible strains and *optrA*-carrying strains. Based on our findings, the mechanism of *optrA*-mediated resistance to oxazolidinones and phenicols obviously does not involve active efflux and the OptrA protein does not confer resistance via efflux like other ABC transporters.

## 1. Introduction

Bacteria have shown an increasing ability to resist the actions of antimicrobial agents, such that the treatment of pathogenic bacterial infections has become a major challenge to public health. Understanding the mechanisms of bacterial resistance is crucial for antibiotic discovery and development. Up to now, major reported drug-resistance mechanisms include drug inactivation by enzymes [1,2], drug efflux pumps [3,4], drug target alteration or protection [5]. Active drug efflux pump has attracted much attention because single multidrug efflux pump can confer resistance to a variety of drugs with different structure and function and may synergize with other drug resistance mechanisms [6]. Active efflux pump genes have been found in Gram-positive and Gram-negative bacteria encoded on the chromosome like *norB* and *tet38* [7], or plasmid like *msr*(A) [8].

Since our first report of the plasmid-mediated *optrA* gene in *Enterococcus faecalis* and *Enterococcus faecium*, which confers transferable resistance to phenicols and last-line antimicrobial oxazolidinone drugs, in China in 2015 [9], there have been reports of the widespread dissemination of this gene among Gram-positive bacteria [10,11,12]. OptrA belongs to the ATP-binding cassette (ABC) transporter F subfamily, which also includes the Vga, Lsa, Sal and Msr proteins, associated with multiple drug resistance [13,14]. ABC-F proteins lack transmembrane domains, and their resistance mechanism, whether through antibiotic efflux or ribosomal protection, remains to be clarified.

In a recent study, Sharkey et al. provided the first direct evidence that Vga and Lsa-type proteins confer resistance by acting directly on the ribosome [15]. However, previous studies showed that *Staphylococcus aureus* and *Staphylococcus haemolyticus* carrying *msr* or *vga* exhibited decreased intracellular accumulation of the corresponding target antibiotics [8,16,17], although this may also result from ribosomal protection [8]. Until now, little has been known regarding the resistance mechanism of *optrA*. Knowing the mechanism of resistance, however, is important to develop new ways to counteract *optrA*-mediated resistance and restore the efficacy of oxazolidinones, one of the last-line antibiotic classes for treatment of MRSA and VRE infections in human clinical medicine.

Here, we report the use of ultra-high performance liquid chromatography-tandem quadrupole mass spectrometry (UHPLC-MS/MS) to investigate the intracellular accumulation of linezolid and florfenicol, as representatives of oxazolidinones and phenicols, respectively, in *optrA*-carrying *Enterococcus faecalis* and *Staphylococcus aureus*. The aim of this study was to find out whether OptrA acts as an antibiotic efflux pump.

## 2. Results

### 2.1. MIC Values

We performed antimicrobial susceptibility testing of the isolates (*E. faecalis* JH2-2, *E. faecalis* JH2-2/pAM401, *E. faecalis* JH2-2/pAM401+*optrA*, *S. aureus* RN4220, *S. aureus* RN4220/pAM401, and *S. aureus* RN4220/pAM401+*optrA*) and found that the MICs remained unchanged as compared to our published data [9]. The MICs of *E. faecalis* JH2-2 carrying plasmid pAM401 with the cloned *optrA* were 64 mg/L for florfenicol and 16 mg/L for linezolid; these were 16-fold and 8-fold higher, respectively, than the MICs of *E. faecalis* JH2-2 and *E. faecalis* JH2-2 carrying only the shuttle vector pAM401. Similarly, the MICs of *S. aureus* RN4220 carrying plasmid pAM401 with the cloned *optrA* were 64 mg/L for florfenicol and 8 mg/L for linezolid, while the corresponding MICs of *S. aureus* RN4220 and *S. aureus* RN4220/pAM401 were at 4 mg/L florfenicol and 2 mg/L linezolid, respectively.

### 2.2. Time Course Study of Accumulation Rules

**Figure 1:**
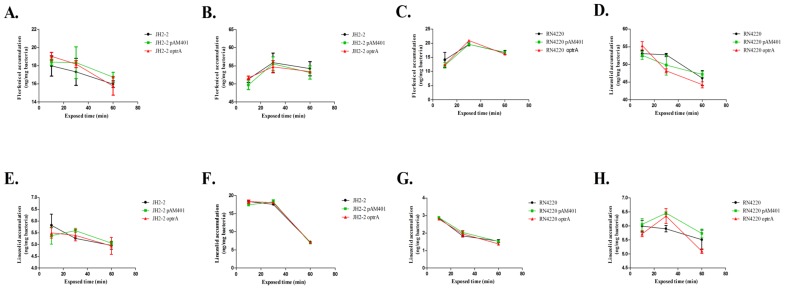
Accumulation of florfenicol and linezolid in the tested bacteria. (**A**) Florfenicol at concentration of 16 mg/L in *E. faecalis*. (**B**) Florfenicol at concentration of 64 mg/L in *E. faecalis*. (**C**) florfenicol at concentration of 16 mg/L in *S. aureus*. (**D**) Florfenicol at concentration of 64 mg/L in *S. aureus*. (**E**) Linezolid at concentration of 2 mg/L in *E. faecalis*. (**F**) Linezolid at concentration of 8 mg/L in *E. faecalis*. (**G**) Linezolid at concentration of 2 mg/L in *S. aureus*. (**H**) Linezolid at concentration of 8 mg/L in *S. aureus*.

**Figure 2:**
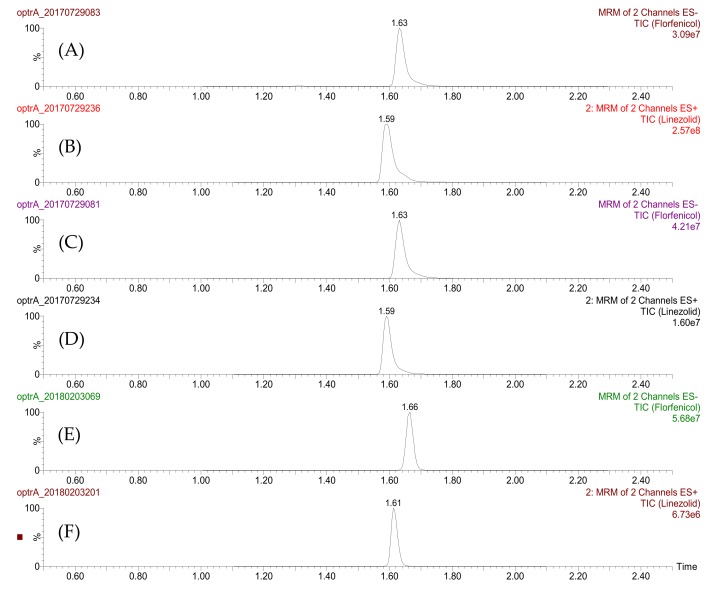
Representative UHPLC-MS/MS chromatograms of florfenicol and linezolid in tested isolates. (**A**) Standard of florfenicol at concentration of 50 μg/L. (**B**) Standard of linezolid at concentration of 50 μg/L. (**C**) Florfenicol at concentration of 64 mg/L in *E. faecalis* JH2-2/pAM401+*optrA* at 60 min. (**D**) Linezolid at concentration of 8 mg/L in *E. faecalis* JH2-2/pAM401+*optrA* at 60 min. (**E**) Florfenicol at concentration of 64 mg/L in *S. aureus* RN4220/pAM401+*optrA* at 60 min. (**F**) Linezolid at concentration of 8 mg/L in *S. aureus* RN4220/pAM401+*optrA* at 60 min.

The results of the time course study of the accumulation of the target compounds are shown in Figure 1 and Table 1. Representative UHPLC-MS/MS chromatograms of florfenicol and linezolid in tested isolates are shown in Figure 2. For *E. faecalis*, the original recipient strain and the two transformant strains exhibited similar trends in accumulation. In the low-dose groups (florfenicol, 16 mg/L; linezolid, 2 mg/L), the highest accumulation was mostly observed at 10 min, and concentrations were slightly lower at 30 min and 60 min, while in the high-dose groups (florfenicol, 64 mg/L; linezolid, 8 mg/L), accumulation commonly peaked at 30 min and then declined again at 60 min. For *S. aureus*, the intracellular concentrations of compounds usually peaked at 10 min in the high-dose florfenicol and the low-dose linezolid experiments, and at 30 min for the low-dose florfenicol and the high-dose linezolid experiments.

Furthermore, analysis of variance (ANOVA) was used to analyze the significance of the accumulation results, and comparisons were carried out between each pair of strains (Table 2). The *p*-values of three comparisons, both in *E. faecalis* and *S. aureus*, were all greater than 0.05.

## 3. Discussion

According to MIC profiles, we selected for this study two incubation concentrations for each antibiotic: 2 mg/L and 8 mg/L for linezolid and 16 mg/L and 64 mg/L for florfenicol. For the exposure time, 10 min was chosen because it is longer than the time required to reach a steady-state concentration, but short enough to minimize metabolic and growth changes [18]. In addition, 30-min and 60-min time points were also included to investigate the trend in the concentrations of the two target compounds. During the 60-min exposure time, no obvious changes were observed for the treated strains in terms of the optical density at 600 nm (OD600).

In contrast to the use of radiolabeled antibiotics and measurement by liquid scintillation counter [8,16,17], we lysed the cells, isolated the target compounds, and determined their concentrations by LC-MS/MS, the “gold standard” for the identification and quantification of small molecules [19,20]. During the sample preparation procedure, we focused on the rapid separation of bacterial cells from culture medium and minimization of cell membrane disruption. Membrane disruption before cell lysis disturbs the internal and external drug balance and may lead to potential inaccuracies in the results. To address this concern, we adopted the silicone oil separation method [18], which has been used to assess the accumulation of over 180 compounds in *Escherichia coli*. This method eliminates the conventional washing step used to remove extracellular drug prior to isolating the cells for lysis, guaranteeing the quality of the quantitative determination of intracellular drug concentrations.

In time course study of the accumulation of the target compounds, these results indicated that no identical accumulation trend was observed in *E. faecalis* and *S. aureus*. It is worth noting that higher dosages of both florfenicol and linezolid resulted in higher accumulation levels in all strains, suggesting that the uptake of the drugs by the bacteria was related to the exposure concentration. However, it was more important to evaluate the difference in accumulation between the susceptible strains and the *optrA*-carrying resistant strains. No significant differences were observed in the patterns of drug accumulation among susceptible strains and *optrA*-carrying resistant strains according to the results of ANOVA. All the above mentioned results clearly demonstrated that the mechanism of *optrA*-mediated resistance to oxazolidinones and phenicols obviously does not involve active efflux and the OptrA protein does not mediate resistance via active efflux like other ABC transporters.

## 4. Materials and Methods

### 4.1. Bacterial Isolates and Growth Conditions

Linezolid- and florfenicol-susceptible and -resistant transformants of *E. faecalis* and *S. aureus*, were obtained from our previous work [9]. They included the susceptible original *E. faecalis* JH2-2 and *S. aureus* RN4220, *E. faecalis* JH2-2/pAM401 and *S. aureus* RN4220/pAM401, which harbor the ‘empty’ shuttle vector pAM401, as well as the resistant *E. faecalis* JH2-2/pAM401+*optrA* and *S. aureus* RN4220/pAM401+*optrA*, which harbor the same vector with a cloned and functionally active *optrA* gene. The tested isolates were grown in brain heart infusion broth and incubated at 37 °C with 200 rpm shaking.

### 4.2. Antimicrobial Susceptibility Testing

The MICs of all susceptible and resistant strains were determined by broth microdilution following the recommendations given in the Clinical and Laboratory Standards Institute (CLSI) documents VET01-S3 and M100-S25 [21,22].

### 4.3. Accumulation and Extraction

Intracellular accumulation assays were performed as described previously with slight modifications [18]. Specifically, 3 mL of overnight cultures of *S. aureus* or *E. faecalis* was added to 300 mL fresh brain heart infusion broth (Beijing Land Bridge Technology Co., Ltd., Beijing, China) and incubated at 37 °C with shaking at 200 rpm until the bacterial growth reached the mid-log phase (OD600 = 0.4). Bacterial cells were harvested at 3200× *g* for 10 min at 4 °C, and the supernatant was discarded. Cell pellets were re-suspended in 40 mL of fresh phosphate-buffered saline (PBS), followed by centrifugation as before and removal of the supernatant. The resulting cell pellets were re-suspended in 6.6 mL PBS, and 650 μL of each cell solution was transferred into a 1.5-mL Eppendorf tube. After equilibrating at 37 °C with shaking for 5 min, the target compounds were added: florfenicol at final concentrations of 16 or 64 mg/L and linezolid at final concentrations of 2 or 8 mg/L. Triplicate samples were prepared for each concentration. Samples were incubated at 37 °C with shaking for 10, 30, and 60 min. After incubation, 600 μL of each culture was carefully transferred into 700-μL precooled (−78 °C) silicone oil (AR20/Sigma High Temperature 9:1, *v*/*v*) to separate the bacterial cells from solution without disturbing the intracellular and extracellular drug equilibrium. Samples with oil were centrifuged at 20,000× *g* for 3 min, and the supernatant and oil were discarded.

For sample lysis, each cell pellet was resuspended in 300 μL water-methanol (2:1, *v*/*v*), and a freeze-thaw cycle of 3 min in liquid nitrogen followed by 3 min in a water bath at 65 °C was performed three times. Lysates were centrifuged at 20,000× *g* for 5 min at room temperature, and the supernatants were transferred into new Eppendorf tubes and centrifuged at 20,000× *g* for 20 min at 4 °C. The supernatant was diluted 100-fold with water-methanol (2:1, *v*/*v*) and vortex-mixed prior to LC-MS/MS analysis.

### 4.4. LC-MS/MS Analysis

Samples were analyzed by a Waters Acquity ultra-performance liquid chromatography system coupled to a Micromass Xevo TQ-S triple quadrupole mass spectrometer (Waters, Manchester, UK) fitted with an electrospray ionization (ESI) source. LC separation was performed using an Acquity BEH C_18_ column (50 mm × 2.1 mm i.d., 1.7 μm particle size; Waters) maintained at 40 °C. The mobile phase was constituted by solvent A (0.1% formic acid in water) and solvent B (0.1% formic acid in acetonitrile) for linezolid, and solvent A (water) and solvent B (acetonitrile) for florfenicol. The flow rate was 0.4 mL/min with a linear gradient under the following conditions: 0–0.2 min, 95% A; 0.2–2.0 min, 95–50% A; 2.0–2.1 min, 50–5% A; 2.1–2.5 min, 5% A; 2.5–2.6 min, 5–95% A; 2.6–4.0 min, 95% A. The injection volume was 5 μL.

The source conditions of the MS/MS system were as follows: capillary voltage, 2.5 kV; source temperature, 150 °C; desolvation temperature, 500 °C; cone gas (N2) flow rate, 50 L/h; desolvation gas (N_2_) flow rate, 800 L/h. Linezolid was analyzed in positive ESI mode, while florfenicol was detected in negative ESI mode. Data were acquired in multiple reaction monitoring mode using MassLynx software v4.1 with the QuanLynx program (Waters, Milford, MA, USA). Optimized MS/MS transitions as well as specific cone voltages and collision energies were as follows: linezolid, cone voltage 30 V, *m*/*z* 338.2 > 296.0 (collision energy 18 eV, transition for quantification), *m*/*z* 338.2 > 195.2 (collision energy 22 eV); florfenicol, cone voltage 48 V, *m*/*z* 355.9 > 336.0 (collision energy 8 eV, transition for quantification), *m*/*z* 355.9 > 184.9 (collision energy, 22 eV). Auto dwell time was applied to ensure that approximately 15 data points were acquired for each chromatographic peak.

### 4.5. Statistical Analysis

Differences in the intracellular drug concentrations were analyzed by SPSS Statistics 21 (IBM Corporation, Armonk, NY, USA), and statistical significance was determined by one-way ANOVA with 95% confidence intervals.

## Figures and Tables

**Table 1:** Concentrations of florfenicol and linezolid in *E. faecalis* and *S. aureus.*

Antibiotic	Concentration (mg/L)	Strains	Concentrations (ng/mg Bacteria)								
			Accumulation Time (min)								
			10			30			60		
Florfenicol	16	JH2-2	17.04	20.21	16.65	14.80	19.97	17.15	16.65	15.82	15.41
		JH2-2 pAM401	17.77	18.48	18.93	19.73	14.85	20.40	17.67	16.75	15.73
		JH2-2 *optrA*	19.72	19.17	18.23	17.66	18.94	17.90	14.40	15.14	17.71
	64	JH2-2	51.40	52.70	49.71	51.28	55.85	60.51	52.32	58.19	52.15
		JH2-2 pAM401	52.04	47.49	49.59	51.91	57.97	56.86	50.74	52.02	56.72
		JH2-2 *optrA*	51.16	51.25	52.63	55.48	51.50	57.20	53.16	53.53	53.45
Linezolid	2	JH2-2	6.50	6.06	4.92	5.33	5.22	5.24	5.16	5.08	4.66
		JH2-2 pAM401	5.07	6.09	4.97	5.76	5.45	5.60	4.91	5.11	5.17
		JH2-2 *optrA*	5.49	5.87	5.13	5.69	5.59	4.91	5.39	4.23	5.21
	8	JH2-2	18.08	18.22	18.73	17.71	17.81	17.08	7.30	7.05	7.12
		JH2-2 pAM401	16.92	17.53	17.81	19.01	18.51	17.32	7.19	7.18	6.38
		JH2-2 *optrA*	17.57	18.40	19.00	17.89	18.29	17.86	6.87	7.88	6.70
Florfenicol	16	RN4220	19.34	12.08	10.96	20.35	18.67	19.76	18.28	15.96	15.95
		RN4220 pAM401	13.14	13.13	10.16	20.48	19.05	19.30	16.29	17.01	17.09
		RN4220 *optrA*	11.77	12.92	12.60	20.72	20.43	21.77	16.56	15.79	16.18
	64	RN4220	54.48	51.51	53.27	52.25	53.61	52.20	44.28	43.82	50.31
		RN4220 pAM401	51.89	51.01	54.80	54.75	49.86	44.81	48.40	45.75	47.46
		RN4220 *optrA*	53.24	57.20	55.49	47.10	48.51	49.02	44.32	42.67	45.67
Linezolid	2	RN4220	2.89	2.96	2.72	1.78	1.89	1.84	1.47	1.43	1.72
		RN4220 pAM401	2.92	2.88	2.85	2.22	1.95	1.97	1.45	1.64	1.47
		RN4220 *optrA*	2.82	2.76	2.83	1.91	1.66	2.29	1.42	1.38	1.34
	8	RN4220	6.12	5.58	6.27	5.73	5.85	6.12	5.00	6.16	5.37
		RN4220 pAM401	6.20	5.64	6.32	6.55	6.42	6.39	6.04	5.58	5.57
		RN4220 *optrA*	5.55	5.72	5.87	5.92	6.31	6.84	5.08	4.97	5.26

**Table 2:** *P*-values of one-way ANOVA of bacterial accumulation of florfenicol and linezolid.

Antibiotic	Concentration (mg/L)	Accumulation Time (min)	Statistical Significance					
			JH2-2 VS JH2-2 pAM401	JH2-2 VS JH2-2 *optrA*	JH2-2 pAM401 VS JH2-2 *optrA*	RN4220 VS RN4220 pAM401	RN4220 VS RN4220 *optrA*	RN4220 pAM401 VS RN4220 *optrA*
Florfenicol	16	10	0.692	0.337	0.554	0.424	0.491	0.905
		30	0.614	0.668	0.938	0.978	0.072	0.075
		60	0.474	0.836	0.365	0.928	0.449	0.401
	64	10	0.288	0.774	0.193	0.739	0.189	0.117
		30	0.924	0.713	0.784	0.279	0.114	0.534
		60	0.647	0.715	0.924	0.606	0.363	0.177
Linezolid	2	10	0.416	0.546	0.823	0.665	0.446	0.251
		30	0.166	0.567	0.370	0.261	0.515	0.604
		60	0.771	0.948	0.723	0.846	0.132	0.174
	8	10	0.076	0.956	0.082	0.813	0.318	0.230
		30	0.160	0.340	0.591	0.060	0.106	0.697
		60	0.551	0.983	0.565	0.503	0.247	0.093
